# Artificial Intelligence in Traditional Chinese Medicine: Unraveling Herbal Medicine’s Mechanisms

**DOI:** 10.34133/research.1224

**Published:** 2026-04-10

**Authors:** Yibo He, Shiyue Wu, Jiayang Li, Shuangyu Chen, Shiliang Chen, Zhezhong Zhang, Beihui He, Yaonan Hong, Chengtao Sun, Guoyin Kai

**Affiliations:** ^1^ The First Affiliated Hospital of Zhejiang Chinese Medical University (Zhejiang Provincial Hospital of Chinese Medicine), Hangzhou, China.; ^2^ Zhejiang Provincial International S&T Cooperation Base for Active Ingredients of Medicinal and Edible Plants and Health, Zhejiang Provincial Key TCM Laboratory for Chinese Resource Innovation and Transformation, Institute of Chinese Medicine Resource Innovation and Quality Evaluation, School of Pharmaceutical Sciences, Jinhua Academy, Zhejiang Chinese Medical University, Hangzhou, China.

## Abstract

Traditional Chinese medicine (TCM), rooted in holistic philosophy, features a “multicomponent, multitarget, multipathway” therapeutic model that has long posed challenges for modern scientific interpretation due to its inherent complexity. Studies elucidating its biological mechanisms have historically relied on correlation-based analytical paradigms. Although artificial intelligence (AI) has been increasingly introduced into TCM research, most current applications remain confined to disease classification, outcome prediction, or herb–target association mining, with limited capacity to reconstruct the underlying biological logic of Zheng (TCM Syndrome) differentiation and formula compatibility. This review systematically elaborates on how network pharmacology serves as a foundational framework, constructing “herb–compound–target–disease” networks that align with TCM’s holistic nature, while AI addresses network pharmacology’s limitations—machine learning streamlines active component screening and ADME/T (absorption, distribution, metabolism, excretion, and toxicity) property prediction, and deep learning decodes spectroscopic data, complex biological interaction networks, and formula synergies. The integrated “computational prediction-experimental validation” workflow, validated across oncology, metabolic diseases, and infectious diseases, has become the gold standard for mechanistic research. Additionally, AI revolutionizes TCM quality control by linking chemical signatures to stable efficacy, integrates multiomics data to construct holistic regulatory networks, and enables translational progress through precision patient stratification, real-world evidence integration, and TCM knowledge graphs that structure fragmented knowledge. With the advancement of technology, generative AI for drug design, large language models for mining ancient texts, and multimodal “life models” promise to deepen integration. Ultimately, AI transcends being a mere tool, translating TCM’s holistic philosophy into modern scientific language, advancing its modernization and internationalization, and offering insights for multitarget drug development in global healthcare.

## Introduction

Traditional Chinese medicine (TCM) is rooted in a profound holistic philosophy that views the human body as an interconnected, dynamically balanced complex system, where disease is considered a disruption of this equilibrium [1,2]. Based on this theory, TCM formulas, especially classic prescriptions, are meticulously designed to intervene simultaneously at multiple biological targets within the body through the synergistic action of numerous chemical components, thereby restoring the system’s balance [3]. This inherent “multicomponent, multitarget, multipathway” therapeutic characteristic is central to the clinical efficacy of TCM, yet it is this very complexity that has long made its material basis and molecular mechanisms of action difficult to clearly elucidate with modern scientific methods [4]. For an extended period, the ambiguity surrounding active ingredients and their targets has been a key bottleneck constraining the modernization and internationalization of TCM.

In stark contrast to the holistic view of TCM, modern Western pharmacological research largely adheres to a reductionist paradigm, centered on a “one target, one drug” development model [1]. This model aims to discover or design compounds that act with high specificity on a single disease-related target. While this strategy has achieved tremendous success in treating many diseases, its limitations become apparent when applied to the analysis of TCM formulas or other complex therapeutic systems characterized by distributed pathway dysregulation. It is fundamentally incapable of capturing the complex synergistic or antagonistic interactions among the multiple chemical components in a formula, interactions that are crucial for the formula’s overall therapeutic effect [1]. Although Western pharmacology has recently evolved toward network medicine, which models diseases as topological modules within the human interactome, this framework remains primarily disease-centric and descriptive, focusing on explaining molecular mechanisms rather than guiding multicomponent therapeutic intervention. In contrast, TCM-oriented network pharmacology (NP) is intervention-driven and purpose-oriented, explicitly designed to model how rational herb combinations coordinately regulate multiple weakly connected but functionally convergent pathways toward a therapeutic equilibrium [5]. Consequently, the traditional research method of screening for active single compounds through trial and error is not only inefficient and resource-intensive but also often reveals only a fraction of the formula’s efficacy, failing to answer the core question of “why this specific combination of ingredients?”. This is particularly true when confronting complex diseases like cancer and diabetes, which are caused by dysregulation across multiple genes and pathways. In such cases, single-target drugs often have limited effectiveness or are prone to resistance, whereas the multitarget intervention strategy of TCM may exhibit unique advantages [4].

Faced with the challenges posed by the complexity of TCM, the scientific research paradigm is undergoing a profound transformation. The rise of systems biology has provided a framework for understanding life activities from a holistic and dynamic perspective. Meanwhile, the explosive development of artificial intelligence (AI) technology has furnished unprecedentedly powerful tools for processing and analyzing the vast and complex data generated by systems biology. The fusion of these 2 fields has given rise to a revolutionary new research paradigm, bringing a glimmer of hope for unveiling the mysteries of TCM [3]. The essence of this shift is not simply to use “modern” technology to validate “traditional” experience but rather to apply a scientific framework that is more aligned with the intrinsic laws of the object of study. The challenge of TCM is rooted in its systemic complexity, and systems biology is precisely the science of studying complex systems. Therefore, the core conflict is not between “tradition” and “science” but between the applicability of 2 different scientific paradigms—reductionism and systems theory—to a specific problem. The intervention of AI is not merely an incremental improvement on existing research but a disruptive necessity. Faced with the massive chemical, biological, and clinical data generated by TCM, traditional analytical methods are overwhelmed [6]. Without the powerful computational capabilities of AI, a systematic analysis of the complex molecular interaction networks produced by TCM formulas would be nearly impossible.

**Fig. 1.**
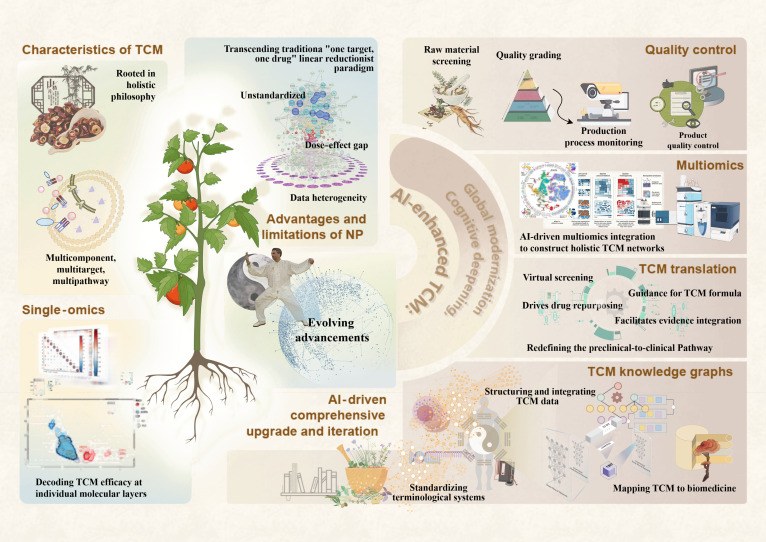
Conceptual framework of artificial intelligence (AI)-driven transformation in traditional Chinese medicine research. This schematic illustrates the paradigm shift from conventional reductionist approaches toward an AI-enabled systems-level understanding of traditional Chinese medicine (TCM). The left panel highlights the intrinsic complexity of TCM, characterized by its holistic philosophy and “multicomponent, multitarget, multipathway” therapeutic mechanisms, along with the limitations of traditional research strategies and single-omics analyses. The middle section presents the integration of AI as a transformative driver, enabling the transition from fragmented data interpretation to comprehensive network-based analysis. The right panel summarizes key AI applications across the TCM research pipeline, including intelligent quality control, multiomics data integration, neural network modeling, and clinical translation through virtual screening and prescription optimization. In addition, AI-powered knowledge graphs facilitate data standardization, semantic integration, and the mapping of TCM theory to modern biomedical frameworks. Collectively, this framework highlights a closed-loop, multidimensional AI–TCM research paradigm that bridges traditional theory with molecular mechanisms and clinical application.

Thus, AI is not merely an “accelerator” of existing workflows but a catalyst that enables the transition from fragmented empirical validation toward system-level mechanistic interpretation. However, a conceptual gap persists: Many current AI models still operate within a reductionist framework, relying on linear classification or regression-based predictions that oversimplify the highly interactive nature of TCM. Transcending these limitations, while recent seminal reviews have systematically categorized AI methodologies within NP [7] or mapped the AI–TCM life cycle from discovery to clinical implementation [8], these efforts have largely remained technology-centered, emphasizing algorithmic applications and data integration pipelines. In contrast, the present review advances a conceptually distinct perspective by focusing on how AI can facilitate the reconstruction and elucidation of the underlying biological logic embedded within TCM theory. Specifically, we propose a multidimensional, closed-loop AI–TCM research paradigm that bridges Zheng-based theoretical constructs with molecular mechanisms (including absorption, distribution, metabolism, excretion, and toxicity [ADME/T] and pharmacological efficacy) and system-level clinical translation through knowledge graphs (KGs) and multiomics integration. Moreover, we advocate a shift from correlation-driven prediction toward causality-informed architectures with enhanced interpretability and multimodal alignment. By repositioning AI as an epistemological bridge rather than merely a computational tool, this framework provides a strategic roadmap for transforming TCM research from empirical observation into mechanistically grounded, AI-enabled precision medicine. A schematic overview of this paradigm shift and conceptual framework is presented in Fig. 1. 

## NP: Mapping the Molecular Action Landscape of Herbal Medicine

NP serves as a pivotal bridge between TCM and modern molecular biology. Its systemic perspective aligns with TCM’s multicomponent, multitarget features, offering a framework to decode holistic therapeutic mechanisms. This section first elaborates on NP’s fundamental principles and “herb–compound–target–disease” networks, then introduces the “network target” concept, and finally addresses key methodological challenges in its application to TCM.

### Fundamental principles: From holistic theory to “herb–compound–target–disease” networks

**Fig. 2.**
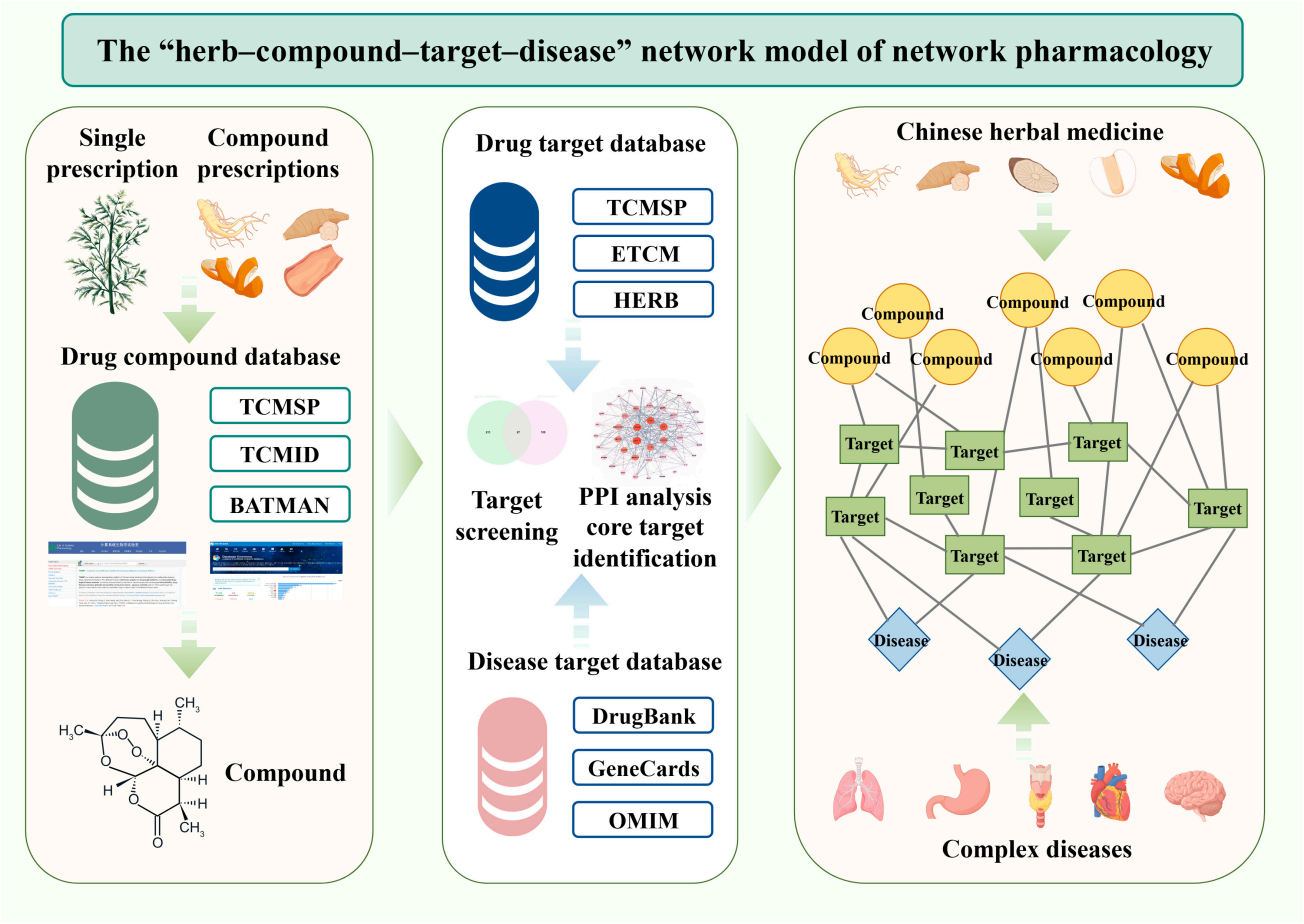
The “herb–compound–target–disease” network model in network pharmacology. This figure illustrates the multilayered network framework underpinning network pharmacology, which is used to decode the mechanisms of traditional Chinese medicine (TCM) prescriptions. Herbs, compounds, targets, and diseases are represented by distinct node shapes (e.g., circles for compounds, rectangles for targets, and diamonds for diseases) and linked through lines that reflect their interactions. The model emphasizes “network targets”—core target nodes associated with disease regulation—highlighted through structural centrality and annotation. The underlying data are sourced from specialized databases such as Traditional Chinese Medicine Systems Pharmacology Database and Analysis Platform (TCMSP), Traditional Chinese Medicine Integrated Database (TCMID), and Bioinformatics Analysis Tool for Molecular mechANism of Traditional Chinese Medicine (BATMAN-TCM) for compound-target mapping, as well as DrugBank, GeneCards, and Online Mendelian Inheritance in Man (OMIM) for disease-related targets. Protein–protein interaction (PPI) analysis further identifies core targets. This integrative model reflects the holistic and systemic nature of TCM, supporting the interpretation of how complex herbal formulas intervene in multifactorial diseases (Table 1).

NP is an interdisciplinary field that represents a paradigm shift from the “one drug, one target” model to a systemic perspective in drug research. In TCM research, its principles align with the multicomponent, multitarget characteristics of herbal medicine, with the core practice being the construction and analysis of “herb–compound–target–disease” networks [1]. As illustrated in Fig. 2, this hierarchical framework visualizes the multilevel mapping from botanical entities to molecular targets, providing a structural basis for interpreting TCM’s holistic efficacy through a systemic lens (Fig. 2).

**Table 1.** Common public databases used in AI-driven TCM research

Category	Database name	Primary content	Role in network pharmacology	References
TCM-specific Databases	TCMSP (Traditional Chinese Medicine Systems Pharmacology Database and Analysis Platform)	Herbal ingredients, ADME (absorption, distribution, metabolism, and excretion) parameters, related targets	Primary source for obtaining active ingredients and initial target information for Chinese herbs	[12]
	TCMID (Traditional Chinese Medicine Integrated Database)	Information on TCM formulas, herbs, ingredients, targets, and related diseases	Provides comprehensive information on formula-ingredient-target relationships	[126]
	BATMAN-TCM2.0 (Bioinformatics Analysis Tool for Molecular mechANism of Traditional Chinese Medicine)	Target prediction for TCM ingredients and pathway enrichment analysis	Predicts ingredient targets and performs functional analysis	[127]
	ETCM (Encyclopedia of Traditional Chinese Medicine)	Comprehensive database of herbs, ingredients, targets, and diseases	An integrative data source supporting network construction	[128]
	HERB2.0 (A high-throughput experiment- and reference-guided database of traditional Chinese medicine)	Experimentally validated herb–target–disease relationships	Provides higher-confidence interaction data	[129]
Target and disease databases	DrugBank	Information on approved drugs, experimental drugs, and their targets	Drug target validation and information query	[49]
	GeneCards	Comprehensive information on human genes, including function and related diseases	Comprehensive retrieval of disease-related gene information	[130]
	OMIM (Online Mendelian Inheritance in Man)	Catalog of human genes and genetic phenotypes	Authoritative source for disease-related genetic genes	[131]
	TTD (Therapeutic Target Database)	Information on therapeutic targets, including successful, clinical trial, and research targets	Query and validation of therapeutic drug targets	[132]
Pathway and interaction databases	KEGG (Kyoto Encyclopedia of Genes and Genomes)	Systematic information on genomes, enzymes, biochemical pathways, etc.	Signal pathway enrichment analysis to understand biological processes involving targets	[51]
	STRING	Known and predicted protein–protein interactions (PPI)	Construction of PPI networks to identify core targets and functional modules	[52]

AI, artificial intelligence; TCM, traditional Chinese medicine; STRING, Search Tool for the Retrieval of Interacting Genes/Proteins

Once the network is constructed, the more critical step is its in-depth analysis to interpret the molecular basis of the herb’s therapeutic efficacy Table 1. Through network analysis, researchers can identify the key players within the system. This approach provides a systematic basis for elucidating TCM’s holistic effects and opens new avenues for its modernization.

### The “network target” concept: A paradigm shift in pharmacological research

In the development of NP, the “network target” concept, proposed by Professor Shao Li’s team [9], represents a landmark core idea. The introduction of this concept marks a profound shift in pharmacological research from focusing on “points” (single target proteins) to “surfaces” (biomolecular networks). It no longer views a drug’s target as an isolated molecule but rather considers the biological network module, composed of multiple molecules associated with a specific disease or syndrome state, as the overall object of intervention [7].

To ensure the scientific rigor and reproducibility of this approach, the field has moved toward standardization with the establishment of the “Network Pharmacology Evaluation Method Guidance” [10]. This guidance provides a robust framework for assessing the reliability and regulatory compliance of NP research, bridging the gap between computational prediction and clinical validation. Recently, the “network target” framework has further evolved into a multidimensional platform that integrates AI and multimodal multiomics technologies [11]. This integration enables the high-throughput decoding of the synergistic mechanisms of complex herbal formulae and the discovery of novel biomarkers, reinforcing AI-empowered NP as a cornerstone for the modernization of traditional medicine [11].

The core task of the “network target” theory involves 2 levels: first, constructing the biomolecular network related to the disease or pattern (concept of “Zheng”); second, based on this network, inferring how a TCM formula or its active components intervene in the disease or pattern by regulating key modules within the network [7]. This idea perfectly aligns with the theoretical essence of TCM: Disease is a manifestation of imbalance in the body’s network, and the goal of treatment is to restore the imbalanced network to a healthy state through the comprehensive action of drugs. Therefore, the “network target” concept has built a crucial bridge for scientifically interpreting the holistic view and pattern differentiation principles of TCM from a modern molecular biology perspective.

### Methodological hurdles: Data heterogeneity, lack of standardization, and the dose–effect gap

Despite its value, NP faces methodological challenges that limit the reliability and reproducibility of its predictions.

First, NP is a “hypothesis-generating engine” that heavily relies on public databases, but the accuracy of its predictions is entirely limited by the quality of the input data. Existing TCM, target, and pathway databases vary widely in source and suffer from heterogeneity, incompleteness, inconsistent standards, and outdated information. Differences in ingredient and target annotations across databases lead to inconsistent analysis results and raise the risk of false-positive or false-negative predictions. Consequently, the entire NP framework operates on a “garbage in, garbage out” principle. The main bottleneck is not the algorithms but the quality and standardization of underlying data. Second, the screening criteria for active ingredients widely used in NP have inherent limitations. Rigid thresholds such as oral bioavailability ≥30% and deep learning (DL) ≥ 0.18 introduce substantial bias [12]. Moreover, compounds with low oral bioavailability or DL may be metabolized into active forms or act as synergists. Simply filtering them out may lead to the omission of critical information [12].

A major overlooked issue is that most NP studies ignore herb dosage information. Dosage compatibility is central to TCM practice and determines a formula’s efficacy and safety. Ignoring dosage means treating the contributions of different herbs in a formula as equal, which is severely disconnected from TCM theory and clinical practice. Studies show that incorporating dosage as a weight markedly alters predicted core ingredients and pathways [13]. This suggests that the vast body of NP literature that does not consider dosage may contain systematic biases, and the reliability of their conclusions needs to be reevaluated. This “dose–effect gap” is one of the most critical fractures that needs to be bridged between computational models and the clinical reality of TCM.

## AI: A Catalyst for Mechanism Discovery and Efficacy Prediction

If NP provides the grand theoretical framework for TCM research, then AI is the powerful engine that drives this framework to operate efficiently. AI, particularly machine learning (ML) and DL, with their exceptional ability to process massive, high-dimensional, and complex data, are fundamentally changing the drug discovery and mechanism research workflow in TCM [14]. However, the deployment of AI methodologies in this context necessitates a rigorous trade-off between predictive potency and biological transparency [15]. While traditional ML models offer high interpretability via feature importance ranking for structured datasets [16], they often lack the capacity to capture the high-dimensional, nonlinear intricacies inherent in TCM omics. In contrast, DL architectures excel at autonomous feature extraction from unstructured data but are constrained by their inherent “black-box” nature [15], necessitating explainable AI (XAI) tools (e.g., SHAP [SHapley Additive exPlanations] and LIME [Local Interpretable Model-agnostic Explanations]) for mechanistic validation [17].

### Applications of ML in the discovery of bioactive components and their targets

**Fig. 3.**
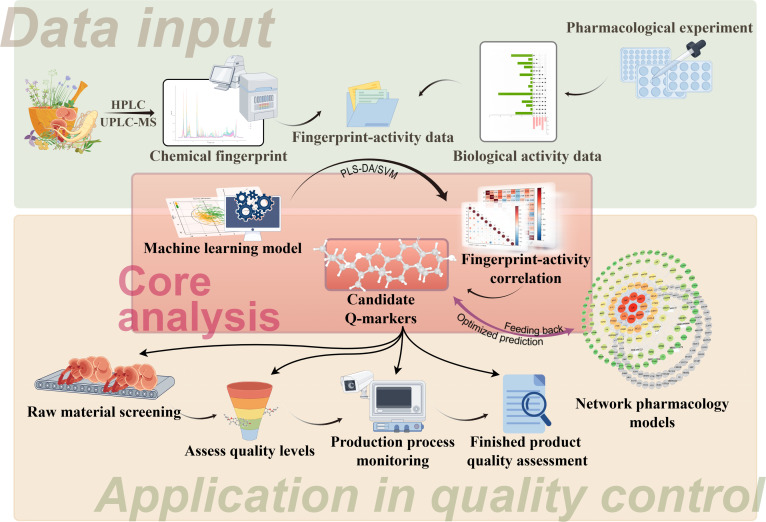
Artificial intelligence (AI)-driven discovery and quality control application path of traditional Chinese medicine (TCM) Q-markers. This schematic illustrates an AI-driven framework for intelligent identification of Q-markers and quality control in TCM. The diagram is structured into 3 coherent modules: The upper green section represents data input, where chemical fingerprints acquired via high-performance liquid chromatography (HPLC)/ultra-performance liquid chromatography–mass spectrometry (UPLC-MS) and bioactivity data derived from pharmacological experiments are integrated into a fingerprint–activity dataset, establishing a multimodal foundation for subsequent analysis. The central red section depicts the core computational analysis, employing machine learning models such as partial least squares discriminant analysis (PLS-DA) and support vector machine (SVM) to decipher underlying correlations between chemical constituents and efficacy, thereby identifying candidate Q-markers. A feedback mechanism is incorporated to iteratively optimize network pharmacology models. The lower orange section demonstrates the application of Q-markers across the quality control pipeline, including raw material screening, dynamic process monitoring, and final product evaluation, constituting a closed-loop control system. Overall, this framework emphasizes the essential role of AI in integrating multiomics data and advancing the standardization of TCM.

In the modernization of TCM, screening bioactive components and identifying their targets are core links to advance the research and development of new TCM drugs and decipher the mechanisms of TCM compound prescriptions. Owing to the complex nature of TCM systems characterized by “multicomponents, multitargets, and multipathways”, traditional experimental screening methods suffer from low efficiency, high costs, and long cycles, making them insufficient to meet the demands of large-scale component mining and target verification. ML, with its robust capabilities in processing structured data, recognizing complex patterns, and ensuring prediction accuracy, has emerged as a pivotal technology to overcome this bottleneck. Among various ML approaches, traditional models such as support vector machines (SVMs) and random forests (RFs) have demonstrated substantial advantages in the preliminary screening of TCM active components, target prediction, and evaluation of pharmacokinetic properties (Fig. 3). By modeling and analyzing the chemical features of compounds, structural features of target proteins, and bioactivity data, these models provide crucial technical support for the transformation of TCM from empirical application to precision research [18].

#### Predictive modeling for active ingredient screening

A single TCM herb or compound prescription typically contains hundreds to thousands of chemical components, among which only a small fraction exhibit definite bioactivity and pharmacological effects. The primary challenge in TCM new drug development lies in efficiently screening potential active molecules from massive compound libraries. ML addresses this challenge by constructing predictive models based on the chemical features of compounds, markedly enhancing screening efficiency and accuracy. Researchers extract multidimensional chemical features of compounds as model input, using compound activity data against specific targets as output to train predictive models, enabling rapid prediction of compound activity.

The core advantages of such models are reflected in 3 aspects. Firstly, they can effectively handle the “highly imbalanced data” issue commonly encountered in drug screening—where active compounds account for an extremely small proportion of the massive library. Through specialized strategies, the models can improve their ability to identify a small number of active samples, avoiding the omission of potential active compounds caused by data bias in traditional methods. Secondly, by integrating multidimensional chemical features, the models can capture the nonlinear relationships between compound structures and activities, and their predictive performance far surpasses that of traditional screening methods relying on a single structural similarity metric. For instance, Xu et al. [19] employed ML to screen 74 active substances and 2,128 herbal prescriptions for adjuvant treatment of gastric cancer from TCM public databases. Subsequent verification confirmed that these prescriptions could inhibit tumor progression through pathway regulation, validating the model’s accuracy. Thirdly, the models exhibit good generalization ability and can be transferred to predict the activity of different types of targets. For example, Huo et al. [20] constructed a predictive model based on the “TCM-target-effect relationship spectrum (TCM-TERS)” and screened 7 compounds with multitarget anti-inflammatory activity from TCM, providing candidate molecules for the multitarget treatment of complex inflammatory diseases.

Simultaneously, chemical signatures—comprising the molecular structure of active pharmaceutical ingredients (APIs), impurity profiles, excipient composition ratios, and particle size distribution (PSD)—serve as essential indicators linking pharmaceutical product quality with therapeutic efficacy. Traditional quality control methods generally analyze these parameters in isolation: API purity via high-performance liquid chromatography and PSD through laser diffraction. However, such isolated analyses fail to account for the synergistic interactions between these chemical attributes, which can substantially influence the product’s efficacy. For instance, slight variations in the PSD of poorly soluble APIs can markedly reduce dissolution rates, directly affecting bioavailability. Furthermore, trace impurities not only breach regulatory standards but may also induce adverse reactions that negate the therapeutic effects of the API [21].

#### In silico pharmacokinetics: Predicting the ADME/T properties of herbal components

Even when a compound exhibits strong in vitro activity, unfavorable ADME/T profiles can severely limit its in vivo efficacy and represent a major cause of drug development failure. Consequently, early prediction of ADME/T properties has become a critical step in AI-driven TCM research, enabling the in silico prioritization of compounds with favorable pharmacokinetic potential and reducing experimental and translational risk. ML models can rapidly estimate ADME/T-related parameters directly from molecular structures, allowing high-throughput virtual filtering of TCM components and focusing experimental resources on the most promising candidates. Mature ML-based platforms, such as SwissADME [22] and ADMETlab 2.0 [23], provide precomputed molecular descriptors and pharmacokinetic predictions that have been widely applied to the ADME/T evaluation of TCM compounds [24].

Beyond molecular structure alone, the translation of chemical composition into stable in vivo efficacy is governed by the complex interplay among multiple chemical signatures, including impurity profiles, excipient composition, and PSD. Conventional quality control methods rely on predefined thresholds for individual attributes and fail to capture nonlinear interactions—such as excipient hydrophilicity affecting release kinetics or crystal polymorphism altering solubility—which often leads to products that meet quality specifications but display variable clinical performance [25]. AI addresses this limitation by integrating multidimensional chemical and process data to model their collective impact on pharmacokinetic exposure and efficacy, transforming threshold-based quality control into predictive, mechanism-informed quality assurance.

ML models further map integrated chemical signatures to efficacy-relevant end points across formulation and manufacturing stages. For example, Hayashi et al. [26] applied ML to predict tablet tensile strength, a determinant of API release consistency, and identified key molecular signatures influencing dissolution performance. In more complex delivery systems, Bannigan et al. [27] demonstrated that ML models integrating chemical and process parameters accurately predicted long-acting injectable release profiles (mean absolute error <5%), substantially outperforming conventional regression approaches.

### DL architectures for advanced pharmacological analysis

With the continuous escalation of data complexity in TCM research, DL models—rooted in neural network architectures—have exhibited unparalleled capabilities in feature learning and pattern recognition. The diverse data modalities inherent in TCM, ranging from high-resolution tongue images and pulse signals to complex “herb–compound–target–disease” interaction networks, have driven the evolution of a specialized AI toolbox. Each DL model, tailored to distinct data structures, addresses unique challenges in TCM pharmacological analysis, thereby unlocking new avenues for decoding the intricate mechanisms of TCM.

#### Convolutional neural networks in the analysis of spectroscopic and microscopic data

Convolutional neural networks (CNNs) efficiently extract spatial hierarchical features from grid-like data. This inherent advantage aligns seamlessly with the visual and spectral data-intensive demands of TCM research, making CNNs a cornerstone technology for TCM quality control, component analysis, and diagnostic objectification.

In the field of herbal material identification and quality control, traditional methods rely heavily on subjective empirical judgment, leading to inconsistencies in authenticity verification and origin tracing. CNNs address this limitation by learning discriminative features from large-scale datasets of Chinese materia medica (CMM) images. For instance, Zhang et al. [28] adapted a CNN approach to microscopic images of CMM powders, successfully distinguishing the cellular structures of easily confused herbs like *Panax ginseng* and *Panax quinquefolius*. Similarly, Zhuang et al. [29] applied a CNN-based system to both tongue diagnosis and CMM slice classification, effectively identifying multiple herbs and detecting quality defects to support standardized quality control.

For spectroscopic data analysis, techniques like infrared and Raman spectroscopy enable nondestructive CMM component detection, but traditional methods require labor-intensive manual feature extraction. CNNs automate deep feature learning from spectroscopic data, eliminating manual engineering and improving accuracy. Hu et al. [30] applied a CNN to Raman spectroscopy of Glycyrrhiza uralensis, automatically analyzing its characteristic components and accurately predicting active content. Jiang et al. [31] adapted a CNN to preprocess infrared spectroscopy data of *Salvia miltiorrhiza* and *Panax notoginseng*, reducing environmental interference and improving component identification accuracy.

Emerging CNN innovations further expand TCM applications. Ni et al. [32] developed a method that enabled simultaneous identification of multiple active component crystal forms in CMM powder microscopic images, supporting research on TCM’s multicomponent material basis. Yang et al. [33] developed a lightweight CNN system for smartphones that enabled real-time on-site detection of CMM, addressing circulation monitoring challenges. The DL model correlated PSD signatures with API dissolution rate in real time, triggering adaptive adjustments to milling speed when PSD deviated by >10% from the optimal range. This reduced batch-to-batch variation in dissolution by 40%, directly improving efficacy consistency. Similarly, Ficzere et al. [34] developed a YOLOv5-based system for film-coated tablets, which simultaneously measured coating thickness and detected defects. The model achieved 98.2% accuracy in defect classification and ±2-μm precision in thickness measurement, ensuring the coating’s barrier function—critical for protecting APIs from degradation and maintaining stable in vivo efficacy.

#### Graph neural networks for modeling complex biological interaction networks

The “herb–compound–target–disease” network—core to TCM NP—is inherently graph-structured: Nodes represent herbs, compounds, targets (proteins/genes), or diseases, while edges denote interactions. Graph neural networks (GNNs) are suited for graph-structured data, capturing complex interactions in TCM biological networks, making them key for analyzing TCM prescription synergies.

GNNs excel in predicting drug–target interactions (DTIs), a critical gap in traditional NP due to database sparsity. GNNs predict unknown DTIs to accelerate active component discovery. Zhao et al. [35] used a GNN approach for TCM prescription recommendation, based on a graph of symptoms, pattern differentiation elements, and herbs. In non-small cell lung cancer research, the model predicted astragaloside IV binds to epidermal growth factor receptor (EGFR). Molecular docking confirmed high binding affinity, comparable to clinical EGFR inhibitors, prioritizing high-potential DTIs for validation. GNNs identify key nodes and functional modules, localizing core components and targets driving TCM efficacy. Hu et al. [36] applied GNNs to analyze the “herb-compound-target” network of heat-clearing/detoxifying herbs for COVID-19. The model identified phillyrin and chlorogenic acid as core components, and angiotensin-converting enzyme 2/transmembrane protease serine 2 as key targets. These modules were linked to viral entry and immune inflammation, aligning with reduced cytokine storms in patients [36]. For complex formulas, GNNs dissect modules matching TCM’s “monarch-minister-assistant-guide” theory: In Liuwei Dihuang Pills, a module was identified and validated to restore kidney function [35].

GNNs also quantify TCM prescription synergies, a challenge for traditional methods. GNNs integrate multidimensional data to model synergies and guide formula optimization. Yang et al. [33] used GNNs for knowledge-driven herb recommendation, incorporating TCM properties and component-target data. In Guizhi Decoction, GNNs calculated synergistic weights between herbs, showing combined inhibition of inflammation, and in vivo tests confirmed reduced edema compared to single components. GNNs also predict optimized synergistic combinations: In Xiaoyao Powder, a new herb pair was identified, and experiments confirmed improved symptoms compared to the original formula [33]. DrugAI, a multiview DL model combining GNNs and CNNs, accurately predicts drug–target activation/inhibition mechanisms and has been experimentally validated, showing robust performance for natural products [37].

### Emerging paradigms: LLMs and multitiered validation strategies

Beyond graph-based and convolutional models, the emergence of large language models (LLMs) facilitates unprecedented multimodal data alignment, bridging ancient texts with modern chemical signatures [38]. However, their deployment in TCM research requires stringent benchmarking to mitigate clinical “hallucinations”. Unlike biomedical corpora with relatively standardized terminologies, TCM knowledge systems are characterized by polysemy in classical language, diachronic synonymy of materia medica, and theory-driven hierarchical inference structures [39]. Consequently, LLM hallucinations in this domain may generate outputs that appear structurally plausible yet disrupt the internal logical coherence of Zheng–pathogenesis–formula–herb reasoning chains [40]. To address this domain-specific vulnerability, we propose a self-verification framework that embeds KG-constrained reasoning and bidirectional logic consistency checks, ensuring that generated interpretations remain aligned with canonical TCM theory and biomedical evidence anchors. Ultimately, to ensure the reliability of AI-driven TCM discovery, a multitiered validation strategy is imperative. This involves integrating “scaffold splitting” for molecular models to evaluate structural generalization [41], alongside robust cross-validation and external clinical cohort testing to bridge the gap between silicon-based predictions and the complex clinical reality of TCM [42].

### The synergy of AI and NP: From prediction to validation

The synergy between AI and NP forms a rigorous closed-loop process from “computational prediction” to “experimental validation”—the gold standard for high-impact traditional Chinese medicine network pharmacology (TCM-NP) research and a key path for translating theory to practice. Pure in silico studies only generate hypotheses about TCM component–biological system relationships; rigorous experiments (in vitro assays, animal models, and clinical trials) are required to confirm these as reliable knowledge [43]. AI’s unique value lies in sifting through massive heterogeneous data to prioritize high-quality, testable hypotheses. By reducing network complexity and quantifying interaction likelihoods, AI focuses experimental efforts.

#### AI-enhanced framework for network mining, positioning, and navigating

To translate TCM’s holistic philosophy into a computable systems biology framework, AI functions as a computational translator that maps macroscopic clinical patterns onto molecular network states [1]. This translation is grounded in a network perturbation–restoration hypothesis, in which pattern differentiation are conceptualized as system-level subnetwork states deviating from homeostasis, and therapeutic efficacy arises from coordinated network reconfiguration rather than single-target modulation [44].

To address the intrinsic limitations of small-sample datasets common in high-quality TCM clinical and multiomics studies, recent advances in AI-driven causal inference have emphasized methods that exploit structural assumptions or shared representations to improve estimation efficiency and robustness. Notably, Bayesian causal learning frameworks have been developed to incorporate prior knowledge and regularize causal graph estimation under limited sample conditions, enabling more reliable mechanistic inference from sparse biomedical data. For example, meta-learning approaches have been proposed to learn personalized causal graph structures by transferring shared causal patterns across related tasks, which enhances causal discovery performance when individual sample sizes are small [45]. In parallel, targeted minimum loss-based estimation and its cross-validated extensions integrate ML models with semiparametric causal estimators, providing doubly robust inference that can better trade off bias and variance in small datasets [46]. Additionally, quasi-experimental designs such as Mendelian randomization leverage genetic instruments to infer causal effects in observational multiomics studies, mitigating unmeasured confounding bias inherent to small-sample observational data [47]. Collectively, these small-sample causal AI methodologies complement the perturbation-restoration paradigm and strengthen the inferential foundation for elucidating TCM mechanisms.

Integrated with advanced AI technologies, TCM-NP research follows a hierarchical 3-stage framework that aligns with Shao Li’s “network target” theory—this theory emphasizes that TCM’s therapeutic effects stem from regulating disease-related molecular network modules, rather than single targets, making it a core paradigm for bridging TCM’s holistic theory with modern systems biology. This framework addresses TCM’s inherent complexity of “multiple components, multiple targets, and systemic regulation” by systematically transforming fragmented data into structured, interpretable networks via AI, laying the foundation for precision TCM research and clinical translation [7,48].

Network relationship mining serves as the framework’s foundational layer, constructing “herb–compound–target–disease–pattern” networks through AI. Natural language processing extracts implicit connections from TCM databases and literature [49,50], while AI integrates these with molecular interaction data to build multilevel networks [51,52]. Single-cell GNNs model cell–cell interactions to reveal cell-specific molecular signatures of hot pattern in gastric premalignant lesions [53]. AI-driven network embedding simplifies high-dimensional data for efficient analysis—Hou et al. [54] used graph embedding to build a human tissue-cell-molecule network, while Tian et al. [55] applied MHADTI for accurate drug-target predictions.

For pattern/disease-related gene prediction, traditional ML methods established the foundation by integrating network topology and clinical data. Recent DL advances, such as GNNs, enhance accuracy: CIPHER-SC fuses single-cell RNA sequencing data with GNNs to predict disease genes in gastric lesions [56], and HGNA-HTI (heterogeneous GNN with attention) prioritizes high-impact nodes in pattern networks [57]. DL models integrate heterogeneous data to boost performance, with transfer learning addressing data scarcity for understudied TCM compounds.

**Fig. 4.**
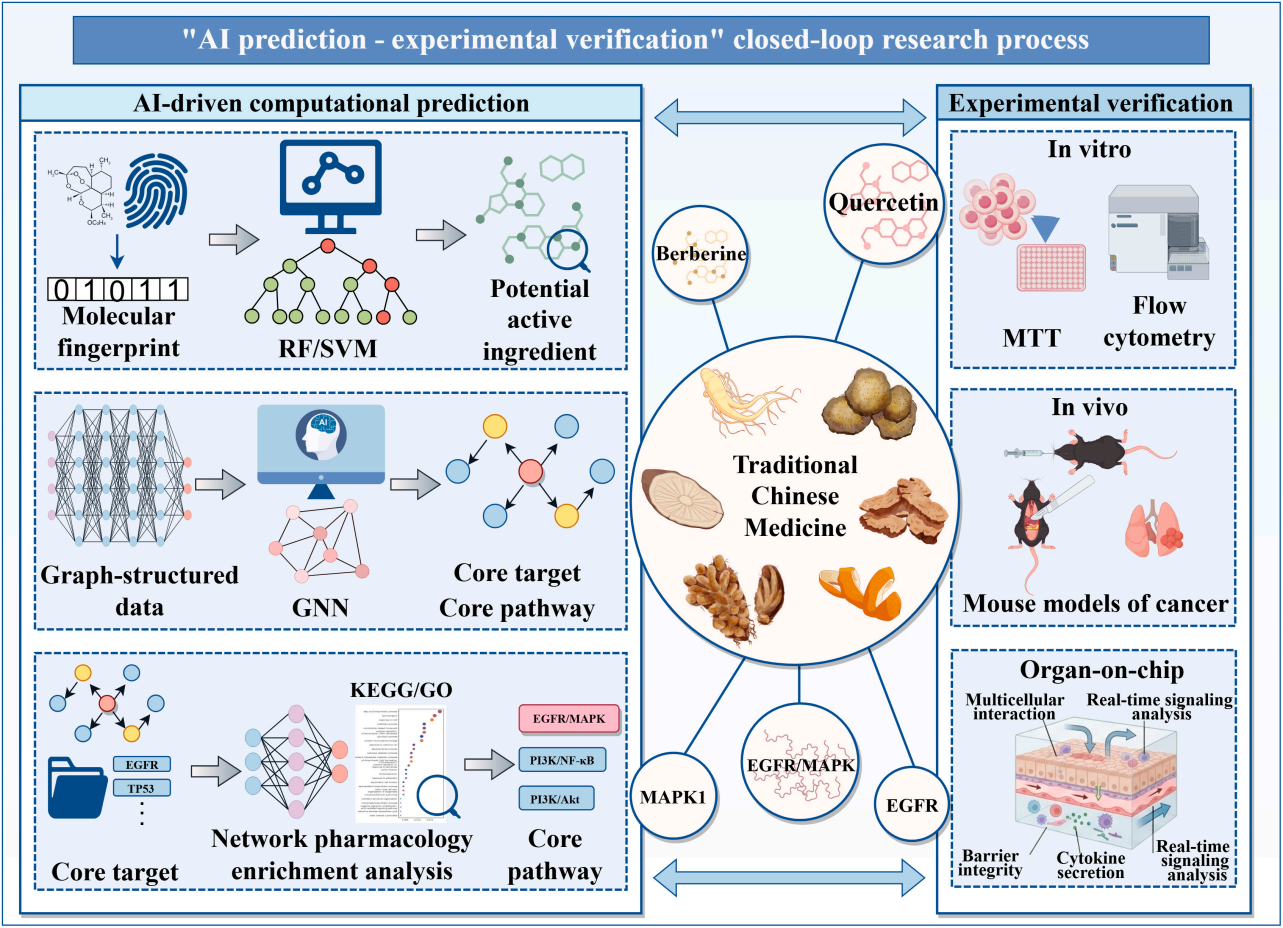
“AI prediction - experimental verification” closed-loop research process. This figure illustrates a golden research paradigm integrating artificial intelligence (AI)-based computational prediction with experimental validation to uncover the mechanisms of traditional Chinese medicine (TCM) formulas. The left section shows the AI-driven prediction module, including active compound screening via machine learning models (e.g., random forest [RF]/support vector machine [SVM]) based on molecular fingerprints and physicochemical properties, core target and pathway identification via graph neural networks (GNNs) analysis of component-target networks, and key signaling pathway determination (e.g., epidermal growthfactor receptor [EGFR]/mitogen-activated protein kinase [MAPK]) via network pharmacology and Kyoto Encyclopedia of Genes and Genomes (KEGG)/Gene Ontology (GO) enrichment analysis. The central part summarizes core compounds, targets, and pathways (e.g., quercetin, EGFR, and MAPK1). The right section presents experimental validation, including in vitro assays (e.g., 3-(4,5-dimethylthiazol-2-yl)-2,5-diphenyltetrazolium bromide [MTT] for cell proliferation and flow cytometry for apoptosis), in vivo cancer mouse models for tumor growth studies, and Organ-on-Chip Modeling (Microphysiological System). Circular arrows represent iterative optimization, where experimental outcomes refine AI models, demonstrating a rigorous “computation-guides-experiment, experiment-validates-computation” loop.

Network target navigating translates mined and positioned networks into practical TCM applications via AI. Within this framework, TCM compatibility is reinterpreted as synergistic network modulation, where multiple herbs collectively optimize system-level perturbations across weakly correlated but functionally convergent pathways [1]. A heterogeneous deep herb-graph method identified reusable COVID-19 herb combinations from 480 Chinese herbs. It enables drug repurposing: ML and NP integrated herb–target and disease–gene networks to find optimal TCM formulas for Alzheimer’s disease and repurpose compound Kushen injection for esophageal cancer. It also supports precision formula recommendation: AI models integrating multimodal data have recommended formulas for chronic atrophic gastritis, with clinical validation confirming their efficacy and alignment with TCM’s treatment principle [43,58]. Similar system-level reasoning has been applied to classical formulas such as Liuwei Dihuang Pill, where AI-based network analysis revealed convergent regulation of the neuro–endocrine–immune network, providing a mechanistic interpretation of the “Kidney-Yin nourishing” principle [59,60]. The above AI-enhanced network research framework provides a systematic methodological support for TCM mechanism exploration, but its practical value ultimately depends on the integration with experimental validation. Recent transfer learning models integrating DL with molecular networks have advanced drug discovery by predicting over 88,000 drug–disease interactions (7,940 drugs, 2,986 diseases) with an area under the curve of 0.93 and identifying potential synergistic drug combinations [61]; while building on these advances, SETComp further predicts cell-specific intervention effects in complex systems, enabling drug repositioning and mechanism discovery [62]. Figure 4 visually presents the “AI prediction - experimental verification” closed-loop research process, which has become the golden paradigm for TCM mechanism research (Fig. 4).

#### Case studies: Integrating AI-NP with experimental validation to elucidate the efficacy of Chinese herbs

The integration of AI and network pharmacology (AI-NP) with traditional experimental validation represents a powerful paradigm for systematically decoding the complex mechanisms of TCM [63]. This approach allows researchers to move from predictive computational models to tangible biological evidence, providing robust insights into the therapeutic efficacy of Chinese herbs [64]. The following case studies exemplify this integrated methodology across a diverse range of pharmacological landscapes. To ensure a comprehensive and rigorous overview, these examples were selected based on 3 strategic criteria: (a) disease and formulation diversity, encompassing high-incidence chronic conditions [65] (e.g., oncology and metabolic disorders) alongside acute pathologies [66] (e.g., COVID-19) and ranging from isolated bioactive molecules to sophisticated multiherb prescriptions; (b) methodological rigor, prioritizing studies that successfully achieve the “silicon-to-bench” closed-loop through high-quality datasets and reproducible AI models [44]; and (c) multiscale validation, selecting cases that represent the current “gold standard” in TCM-NP by integrating computational evidence (e.g., molecular docking and MD simulations) with experimental verification (in vitro assays and in vivo animal models) [3,67]. Such stringent selection ensures that the discussed cases are not only representative of the field’s frontier but also possess the generalizability required to guide future clinical translation. The following case studies exemplify this integrated methodology across a diverse range of diseases, from cancers and metabolic disorders to infectious and inflammatory conditions.

An important area of application has been in oncology. For instance, a study on Zuojin Pill for hepatocellular carcinoma identified quercetin and berberine as core components, targeting key pathways. Experimental validation confirmed the formula’s efficacy and the accuracy of the computational predictions. Similarly, research on Xiaojianzhong Tang for gastric cancer identified multiple active ingredients and core targets. In vitro experiments confirmed the formula’s inhibitory effect on cancer cells and the downregulation of key predicted targets [68]. An AI-driven study on Polygonati Rhizoma for gastric adenocarcinoma identified diosgenin as a core component, predicting its stable binding to key targets. This was corroborated by simulations and confirmed through in vitro inhibitory assays [69].

This integrated strategy has also been highly effective in elucidating the mechanisms of TCM for metabolic diseases. For instance, a study on quercetin for treating diabetic wounds combined computational and experimental methods to identify relevant pathways, with results validated in a disease model [70]. A diabetic rat model experimentally validated these predictions, showing that quercetin markedly reduced inflammatory cytokines and confirming the role of the phosphatidylinositol 3-kinase–protein kinase B pathway [70]. Research on *Berberis integerrima* for diabetic nephropathy utilized NP and ML to pinpoint key targets, confirmed via molecular simulations [71]. Another study on the formula Ganluo Huoxiang for the same condition revealed its core mechanism, which was subsequently verified in a cellular model [72]. Furthermore, this approach has been extended to other metabolic disorders, including studies on fenugreek for diabetes, oleanolic acid for obesity, and Yinchen Wuling Powder for hyperlipidemia [73].

The approach has also clarified treatments for inflammatory, fibrotic, and infectious diseases. Studies on formulas such as Shexiang Baoxin Pill for atherosclerosis and Buyang Huanwu Decoction for myocardial fibrosis predicted relevant pathways and targets, with efficacy confirmed in experimental models. Applications extend to infectious diseases, as seen in research on Chufeng Qingpi Decoction for schistosomiasis.

Further examples demonstrate the broad applicability of this research paradigm. For instance, Wensan tincture was predicted to act through the mitogen-activated protein kinase signaling pathway in treating solitary pulmonary nodules [74]. Cepharanthine was investigated for COVID-19, where MD simulations validated that angiotensin-converting enzyme 2 was the most critical target, binding stably with cepharanthine [75]. Additionally, a comprehensive analysis of Haizao Yuhu Decoction for hyperthyroidism identified key active components and confirmed its efficacy in inhibiting pathological angiogenesis and modulating immune responses [76].

Table 2 summarizes these representative case studies, highlighting the computational methods and key predictions.

**Table 2.** Representative case studies demonstrating AI-enabled network pharmacology from mechanistic discovery to clinical translation in TCM

TCM compound/system	Disease background	Calculation methods (AI component)	Predicted key active components	Predicted key targets/ clinical outcomes	References
Zuo Jin Wan (ZJP)	Hepatocellular carcinoma (HCC)	NP (ADME screening), pathway enrichment analysis	Quercetin, berberine	MAPK1, PIK3CA, EGFR; PI3K/NF-κB, EGFR/MAPK signaling pathways	[133]
Xiao Jian Zhong Tang (XJZ)	Gastric cancer (GC)	NP, PPI network analysis, molecular docking	Quercetin, licoagrocarpin	IL-6, PTGS2, MMP9, HMOX1	[68]
Polygonati Rhizoma	Gastric adenocarcinoma (STAD)	AI-driven NP (RF, SVM), molecular docking, molecular dynamics (MD) Simulation	Diosgenin, baicalein, β-sitosterol	AKT1, TP53, VEGFA	[69]
Chu Feng Qing Pi Tang (CQD)	Schistosomiasis	LASSO, RF, SVM-RFE, PPI network analysis, molecular docking, molecular dynamics simulation	Wogonin, kaempferol, luteolin, quercetin	TP53	[134]
Wen San Ting (WST)	Pulmonary nodule (SPN)	PPI network analysis, molecular docking	Quercetin	AKT1, TP53, MAPK1, MAPK3	[74]
She Xiang Bao Xin Wan (SBP)	Atherosclerosis (AS)	PPI network analysis, molecular docking	Chenodeoxycholic acid, ursodeoxycholic acid, cinnamaldehyde, ginsenoside Rb1, etc.	MAPK3, AKT1, STAT3	[135]
Cefapirin (CEP)	Coronavirus disease 2019 (COVID-19)	PPI network analysis, molecular docking, molecular dynamics simulation	Quercetin	ACE2, PI3K-Akt signaling pathway	[75]
Hai Zao Yu Hu Tang (HYD)	Hyperthyroidism	PPI network analysis, molecular docking, molecular dynamics simulation	Quercetin, β-sitosterol, naringenin, kaempferol, wogonin	PTPN11, PIK3CD, EGFR, HRAS, PIK3CA, AKT1, SRC, PIK3CB, PIK3R1	[76]
Quercetin (QCT)	Chronic nonhealing diabetic wounds (DW)	PPI Network analysis, molecular docking, molecular dynamics simulation	Quercetin	IL-6, EGFR, SRC, TNF, AKT1, JUN, MMP9; AGE-RAGE, IL-17, PI3K-AKT, TNF, HIF-1, VEGF and other signaling pathways	[19]
Berberine	Diabetic nephropathy (DN)	PPI network analysis, molecular docking, molecular dynamics simulation	Alkaloids (armepavine, berberine, glaucine, magnoflorine, reticuline, quercetin)	ICAM1, PRKCB, IKBKB, KDR, ALOX5, VCAM1, SYK, TBXA2R, LCK, F3 genes	[71]
*Astragalus membranaceus* (AM)	Idiopathic pulmonary fibrosis (IPF)	PPI Network analysis, molecular docking, molecular dynamics simulation	Astragaloside III, (R)-isomucronulatol, astragaloside I, paeoniflorin, β-sitosterol	AKT1, HSP90AA1, VEGFA	[136]
Bu Yang Huan Wu Decoction (BYHWD)	Myocardial fibrosis (MF)	PPI network analysis, molecular docking	Quercetin, luteolin, peonidin, ellagic acid, baicalein	IL-17 signaling pathway	[137]
*Trigonella foenum-graecum*	Diabetes mellitus	PPI network analysis, molecular docking, molecular dynamics simulation	Diosgenin, luteolin, quercetin	ESR1, CAV1, VEGFA, TP53, CAT, AKT1, IL-6, IL-1	[138]
Oleanolic acid (OA)	Obesity	PPI network analysis, molecular docking, molecular dynamics simulation	Oleanolic acid	PPARG, PPARA, MAPK3, NR3C1, PTGS2, CYP19A1, CNR1, HSD11B1, AGTR1	[73]
Yin Chen Wu Ling San (YCWL)	Hyperlipidemia	PPI network analysis, molecular docking, molecular dynamics simulation	Quercetin, isorhamnetin, taxifolin, demethoxycapillarisin, artemisinin A	AKT1, IL-6, VEGFA, PTGS2	[139]
Ganhua (LLF)	Diabetic nephropathy (DN)	PPI network analysis, molecular docking, molecular dynamics simulation	Salidroside (Sal), apigenin (Api), torularic acid (TA)	TNF	[72]
AI-assisted TCM diagnostic system	Diabetes, gastrointestinal disorders (clinical cohorts)	ML-based clinical classification models trained on EMR and symptom data	n/a	Improved diagnostic accuracy and pattern differentiation in real clinical settings	[140]
AI-enabled TCM pattern recognition system	Multicenter TCM pattern diagnosis	Machine learning-based pattern classification	n/a	Enhanced consistency of pattern differentiation across clinicians (with noted cross-center limitations)	[141]
AI-assisted TCM drug R&D pipeline	TCM drug development (safety/efficacy assessment)	AI-based ADME/T prediction, safety screening, regulatory support	Multiple candidate components	Facilitated translational decision-making from preclinical to clinical phases	[101]

AI, artificial intelligence; TCM, traditional Chinese medicine; NP, network pharmacology; ADME, absorption, distribution, metabolism, and excretion; ADME/T, absorption, distribution, metabolism, excretion, and toxicity; ML, machine learning; SVM, support vector machine; RF, random forest; EGFR, epidermal growth factor receptor; PI3K, phosphatidylinositol 3-kinase; AKT1, protein kinase B1; MAPK3, mitogen-activated protein kinase 3; IL, interleukin; STAT3, signal transducer and activator of transcription; n/a, not applicable; ACE2, angiotensin-converting enzyme 2; LASSO, least absolute shrinkage and selection operator; RFE, recursive feature elimination; EMR, electronic medical record

### Critical limitations and risks of AI-driven TCM research

The paradigm shift toward AI–driven TCM research represents not merely a pursuit of computational efficiency but a biological necessity imposed by the intrinsic complexity of herbal systems. The multicomponent, multitarget, and multipathway characteristics of TCM generate a high-dimensional combinatorial space that exceeds the analytical capacity of conventional reductionist approaches. Advanced AI architectures, including GNNs and transformer-based models, are uniquely capable of modeling such nonlinear and hierarchical relationships. However, the same properties that confer modeling power also introduce systemic methodological risks that must be critically addressed.

A central limitation arises from the “black-box” nature of many high-performing DL models, which often trade mechanistic interpretability for predictive accuracy, a challenge that has been widely discussed in the context of medical AI explainability and trustworthiness [16,77]. In the context of TCM, where therapeutic efficacy is rooted in the biological logic of synergy and coordinated pathway modulation, models that yield accurate predictions without explicating underlying network perturbations remain of limited translational value. This challenge is further compounded by the “small-sample, high-dimensional” data structure typical of TCM research, in which multiomics features are abundant while high-quality, standardized clinical annotations remain scarce, substantially increasing the risk of overfitting and spurious associations [78,79].

Another critical bottleneck lies in multimodal data alignment. AI-driven TCM studies increasingly integrate heterogeneous data sources—including classical medical texts, chemical fingerprints, omics profiles, phenotypic readouts, and real-world clinical metrics—each characterized by distinct scales, noise structures, and semantic frameworks [80]. Inadequate harmonization across modalities may propagate systematic bias into model training, undermining reproducibility and generalizability. Beyond technical concerns, ethical and regulatory challenges emerge when AI-assisted predictions are extended toward clinical decision-making, particularly with respect to data privacy, algorithmic bias, and accountability in medical inference.

## Multiomics Frontiers: AI-Driven Integration of System-Level Views

To understand the mechanisms of action of Chinese medicine more comprehensively and deeply, research has moved from focusing on molecular interactions at a single level to the “Multiomics” era, which integrates data from multiple biological levels. AI plays an indispensable role in this frontier field as a tool for data integration and pattern discovery.

### Single-omics technologies: Decoding TCM efficacy at individual molecular layers

Single-omics technologies serve as the foundational building blocks for dissecting the molecular mechanisms of TCM, each focusing on a distinct layer of biological information to unravel how TCM’s active metabolites interact with biological systems. Epigenomics, for instance, explores reversible chemical modifications to DNA and associated proteins—such as DNA methylation and histone acetylation—that regulate gene expression without altering the underlying genetic sequence. This layer is critical for understanding TCM’s long-term regulatory effects: Ming et al. [81] showed that curcumin exerts anticancer effects by inhibiting DNA methyltransferase and histone deacetylase activity, highlighting TCM-induced epigenetic remodeling. Such findings link TCM’s pharmacological actions to epigenetic remodeling, a mechanism often overlooked in traditional reductionist studies.

Genomics, by contrast, leverages high-throughput sequencing and genetic profiling to identify TCM’s target genes and genotype-phenotype associations. It plays a pivotal role in biomarker discovery and patient stratification, aligning TCM’s holistic principles with precision medicine. Xu et al. [82] identified Nfkb1, Stat1, and Ifnrg1 as core targets of Gegen Qinlian Decoction, linking these genes to its antidiabetic effects. Databases like the Gene Expression Omnibus [83] and The Cancer Genome Atlas [84] provide large-scale genomic datasets that enable AI algorithms—such as SVM and RFs—to mine disease-specific target signatures, bridging genomic variation with TCM’s therapeutic outcomes.

Proteomics and metabolomics further extend single-omics insights by linking genomic information to functional phenotypes. Proteomics characterizes protein expression and interactions central to TCM-modulated pathways. Xu et al. [85] identified serum biomarkers PI3, CCL22, and interleukin-12B that correlate with TCM responses in psoriasis. This study highlights how proteomics, paired with AI, can validate TCM’s efficacy at the protein level. Metabolomics profiles small-molecule metabolites reflecting TCM-induced physiological changes. Wu et al. [86] found that Shaoyao Decoction modulates signal transducer and activator of transcription 3, interleukin-1β, and protein kinase B1 through key metabolites such as quercetin and baicalin. Databases such as the Human Metabolome Database [87] and Kyoto Encyclopedia of Genes and Genomes [51] support these analyses by providing comprehensive metabolite annotations and pathway mappings.

Spatial omics links systemic TCM effects to localized molecular responses. Spatial technologies such as Visium and GeoMx map molecular distributions within tissues. Laubscher et al. [88] used spatial transcriptomics with GNNs to reveal tissue-specific actions of ephedrine in asthma. This spatial resolution, when combined with AI’s ability to model complex spatial relationships, transforms static molecular observations into dynamic, tissue-specific mechanisms, a critical step toward understanding TCM’s holistic efficacy.

### Multiomics integration: Constructing holistic networks of TCM action

Multiomics integration, supported by AI, captures the interconnected multimetabolite and multitarget actions of TCM beyond what single-omics can reveal. Beyond identifying associations, AI can also support causal inference, revealing how specific TCM components perturb molecular networks to drive therapeutic outcomes. Traditional approaches, such as NP or static correlation analyses, fail to account for cross-layer crosstalk. AI integrates heterogeneous genomics, proteomics, metabolomics, and spatial omics data to construct system-level TCM network models. ML algorithms are particularly effective at identifying linear and nonlinear patterns across omics layers. RFs, for example, have been used to integrate genomic and proteomic data to predict TCM’s anticancer targets, leveraging their ability to handle high-dimensional data and reduce overfitting. Chen et al. [89] used RF and SVM to integrate proteomic and metabolomic data, identifying metabolites interacting with the neurodegeneration-related target glycogen synthase kinase 3 beta. These AI-enabled multiomics strategies provide a systems-level framework for studying TCM mechanisms, moving from correlation to causality to uncover the biological logic of TCM interventions.

DL models further enhance multiomics integration by automatically learning high-order features that elude traditional methods. CNNs excel at processing high-dimensional data from multiple omics sources: Liu et al. [90] used a CNN-based multiomics fusion model to predict TCM-derived Parkinson’s disease drug candidates, outperforming single-omics methods. GNNs, meanwhile, are uniquely suited for modeling networked data—such as metabolite-target interactions—by treating omics entities as nodes and their interactions as edges. Duan et al. [91] developed HTINet2, a GNN framework that extracts metabolite-target interaction patterns from multiomics data [91].

**Fig. 5.**
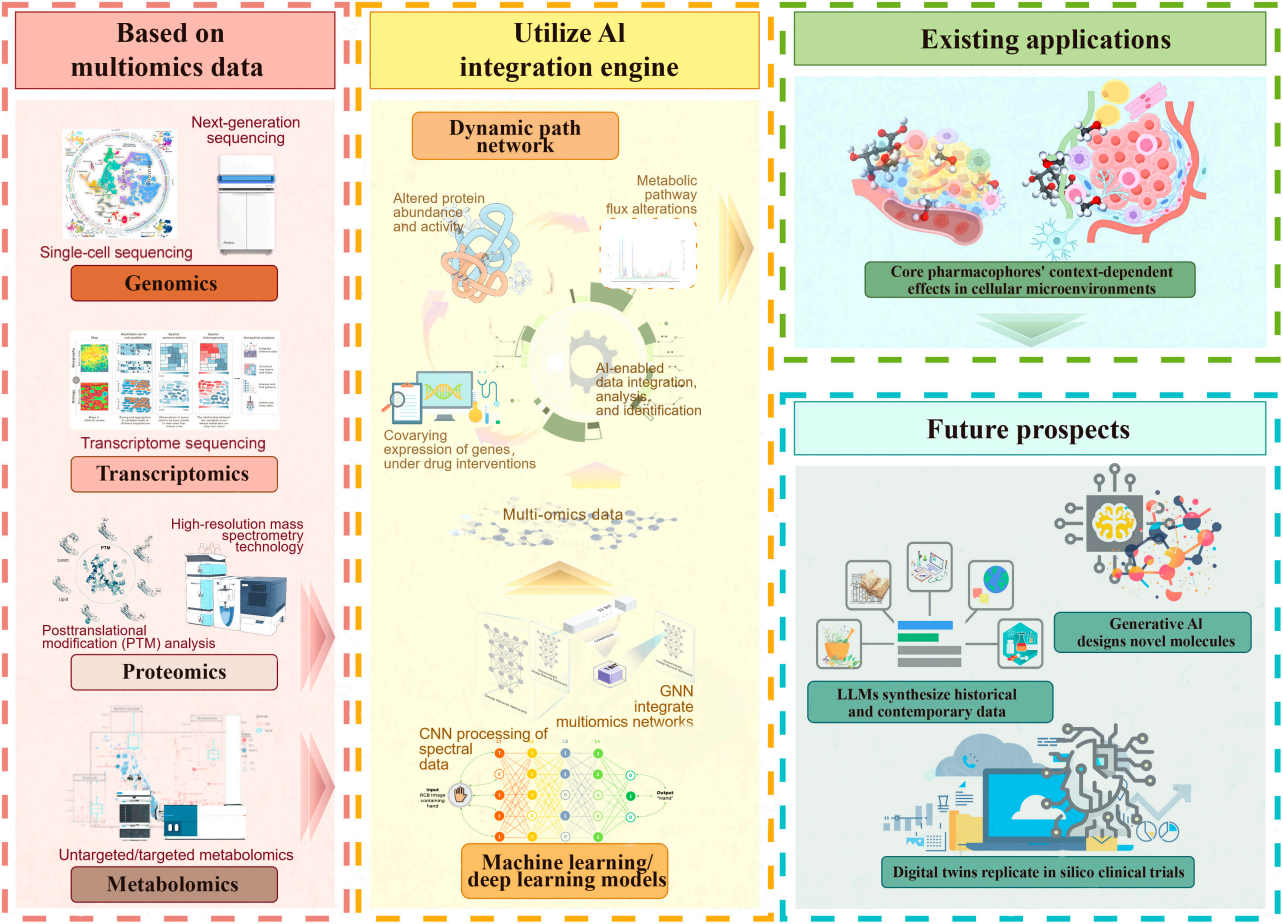
Integration of multiomics and artificial intelligence (AI) for deciphering systemic mechanisms of traditional Chinese medicine (TCM) and future perspectives. This figure presents the methodological framework and future development direction of integrating multiomics and AI in the analysis of the pharmacological mechanisms of TCM systems. The left module provides a multilevel data foundation from the molecular to the system level through key technologies in genomics, transcriptomics, proteomics, and metabolomics. The central module serves as the core AI integration engine, relying on machine learning/deep learning methods such as convolutional neural network (CNN) and graph neural network (GNN) to dynamically analyze system responses such as gene coexpression, protein function regulation, and metabolic network reconstruction under drug intervention, thereby constructing explainable pharmacological pathway models. The upper right corner shows current representative applications, such as the study on the context-dependent mechanism of the pharmacophore of quercetin in the cellular microenvironment. The future directions are proposed at the lower right corner, including generative AI-assisted molecular design, the integration of traditional knowledge and modern data by large language models, and virtual clinical trials based on digital twins. This reflects the transformation from reductionism to the systems biology paradigm and highlights the driving role of AI in the modernization research of TCM.

Cross-modal fusion algorithms harmonize diverse omics modalities for integrated analysis. Joint embedding methods such as deep canonical correlation analysis create shared feature spaces to uncover cross-omics regulatory patterns. Lian et al. [92] applied deep canonical correlation analysis for cross-modal feature extraction, a strategy adaptable to multiomics integration. Generative models such as GANs and VAEs support multiomics synthesis by generating molecular structures and learning shared latent representations. The multimodal knowledge graph-based feature extraction neural network framework, for instance, integrates neural networks with multimodal KGs to model drug–chemical–target interactions, optimizing TCM formulation design by aligning multiomics data with clinical outcomes. Figure 5 provides a comprehensive visualization of this integration, illustrating how cross-layer crosstalk is captured to move beyond simple correlation toward a causal understanding of how TCM formulas reorchestrate biological systems (Fig. 5).

## From “Computation” to “Clinic”: The Translational Pathway and Real-World Challenges of AI-Empowered TCM

AI is reshaping the translational pathway from preclinical research to clinical application in TCM. By bridging computational hypotheses with viable clinical candidates, AI addresses long-standing challenges such as high costs, extended timelines, and low success rates in TCM drug development and evidence generation. This transition is marked by AI’s role in enhancing the efficiency of identifying active compounds and optimizing multiherb formulations preclinically, while innovating clinical trials through precision patient stratification and real-world evidence integration postclinically. These applications represent demonstrated successes where AI has already begun reshaping the traditional TCM research paradigm. However, substantial future potential remains, particularly in areas such as personalized herbal medicine formulation and the use of digital twins to predict herb–drug interactions in silico, which could revolutionize the field.

### Redefining the preclinical-to-clinical pathway with AI

AI is reshaping the preclinical-to-clinical pathway by bridging computational predictions with clinically actionable candidates. A core application lies in large-scale virtual screening, where AI-driven models enable efficient identification of promising active compounds from extensive libraries—including herbal-derived molecular databases. Using advanced architectures—such as equivariant networks and AlphaFold2-based cofolding—AI predicts ligand conformations and binding poses with accuracy that exceeds traditional screening [93]. Such capabilities are critical given the exponential growth of commercial compound collections, which makes comprehensive experimental screening computationally infeasible.

AI also drives drug repurposing, a strategy that repurposes existing approved drugs for new therapeutic uses by analyzing large-scale biomedical datasets. This approach reduces the time and cost associated with drug discovery by uncovering previously unrecognized therapeutic properties of approved drugs. AI further supports repurposing by simulating clinical outcomes from real-world data and classifying drugs via omics-based DL. AI-powered phenotypic screening helps reveal mechanisms of action, strengthening repurposing pipelines.

Furthermore, AI guides the optimization of multiherb formulations by integrating ADME/T prediction and synergy analysis. GNNs and transformer models predict molecular properties to optimize synergy while controlling toxicity. HistoCell, a weakly-supervised DL model, infers single-nucleus cell types and states from histology images, enabling identification of spatial biomarkers for cancer diagnosis and precision therapy [94]. These models address the complexity of multicomponent systems by integrating multiomics data and chemical knowledge, ensuring that formulations balance efficacy and safety. This data-driven workflow prioritizes high-value candidates for experimental validation, improving the overall efficiency of preclinical-to-clinical translation.

Despite these computational advancements, a critical gap remains between in silico predictions and clinical reality [18]. While current successes are primarily confined to the preclinical discovery phase—such as the identification of lead compounds through AlphaFold2-based structural predictions [95]—the practical clinical applicability of AI-optimized multiherb formulations is often constrained by the intrinsic complexity of TCM matrices. Unlike synthetic drugs, herbal ingredients undergo extensive metabolic transformations in vivo, a process that current AI models (often trained on simplified chemical datasets) are not fully equipped to capture [96]. Furthermore, the persistent “garbage in, garbage out” challenge remains a bottleneck; the lack of standardized bioactivity data for secondary metabolites across different herb origins poses a substantial threat to the reliability and reproducibility of AI-driven predictions.

### Innovating clinical trials and evidence generation in TCM

A major advancement is precision patient stratification, essential for TCM where efficacy varies across heterogeneous populations defined by pattern differentiation. AI algorithms, leveraging ML and LLMs, can integrate multiomics data (genomic and metabolomic) with clinical information (symptom patterns, tongue diagnosis features, and treatment history) to identify distinct patient subgroups most likely to respond to specific TCM interventions. Such stratification reduces intergroup variability and increases trial efficiency, addressing the lack of fine-grained patient classification in traditional TCM trials.

Beyond patient stratification, AI drives the discovery of novel, objective biomarkers from complex TCM datasets, a critical step toward quantifying TCM efficacy—a long-standing barrier to evidence-based recognition. Traditional TCM efficacy assessment relies heavily on subjective symptom evaluations, but AI can analyze high-dimensional data (imaging, laboratory indices, and wearable device data) to identify latent, quantifiable biomarkers that correlate with TCM treatment responses [97]. These biomarkers offer objective end points that strengthen trial evidence and improve reproducibility.

AI also facilitates the integration of real-world evidence from electronic health records (EHRs) into TCM evidence generation, a practice that enhances the external validity of TCM research. Analyzing deidentified EHR data allows AI to continuously monitor TCM effectiveness across real-world populations [98]. This integration not only validates findings from controlled trials but also creates a feedback loop: Real-world data insights refine trial design for future studies, while trial results inform more precise TCM clinical practice, aligning with the “clinical trials informed framework” that emphasizes effectiveness evaluation and postdeployment monitoring [99].

Beyond these advancements, the translation of AI-generated evidence into clinical practice must navigate a complex regulatory and interpretability landscape. Currently, the “black-box” nature of complex AI algorithms remains a barrier to clinical trust [100], as TCM practitioners require transparency to align computational results with traditional pattern differentiation (Zheng). Furthermore, the lack of standardized regulatory frameworks from global health authorities (e.g., National Medical Products Administration and Food and Drug Administration) specifically for AI-driven TCM evidence limits its formal integration into drug registration and clinical guidelines [101]. Bridging this gap will require the development of “explainable AI” (XAI) models that prioritize biological and theoretical plausibility alongside statistical rigor [102], ensuring that AI serves as a transparent and reliable tool for the future of evidence-based TCM.

### Practical implementation of AI in TCM research: Actionable strategies and lessons learned

Although AI has substantially advanced the theoretical integration of TCM with systems biology, its translational impact depends on practical implementation. To move beyond abstraction, we summarize a tripartite implementation framework distilled from both successful and failed AI–TCM applications (Table 3).

**Table 3.** Common pitfalls and actionable solutions in AI-driven TCM research

Stage	Common pitfall	Practical solution	Recommended tools
Data	Heterogeneous pattern labels	FAIR + ontology	TCM-Ontology, OMOP
Model	Black-box predictions	XAI / logic-guided NN	SHAP, GNNExplainer
Validation	No experimental closure	Organ-on-chip + retrospective	Lung chip, gut chip

AI, artificial intelligence; TCM, traditional Chinese medicine; XAI, explainable AI; FAIR, findable, accessible, interoperable, reusable; SHAP: SHapley Additive exPlanations; OMOP, Observational Medical Outcomes Partnership

#### Data standardization as the prerequisite for reliable modeling

AI performance in TCM is fundamentally constrained by data quality. We recommend building standardized, structured, and dynamically updated databases that reflect the multicomponent–multitarget nature of TCM prescriptions, following FAIR (findable, accessible, interoperable, reusable) principles. This directly addresses the reproducibility issues commonly arising from heterogeneous pattern labels, inconsistent chemical annotation, and unstructured clinical records.

#### Model interpretability to enable clinical trust

For clinical translation, interpretability is as critical as accuracy. White-box and gray-box models—such as symbolic regression and logic-guided neural networks—provide mechanistic transparency while retaining predictive power. For example, AI-guided protein structure prediction (e.g., AlphaFold2) has accelerated the identification of bioactive TCM-derived compounds by offering interpretable structural hypotheses for experimental validation. Likewise, explainable network models have mapped pattern (e.g., Cold/Heat) onto immune–metabolic regulatory modules, strengthening mechanistic understanding and clinical confidence.

#### Closed-loop validation to ensure biological relevance

We advocate a 3-stage validation pathway integrating in silico prediction, organ-on-chip modeling, in vivo validation, and retrospective clinical analysis. This closed-loop strategy has proven effective in AI-assisted early detection of gastric mucosal lesions, where computational predictions were corroborated by multimodal clinical and histopathological evidence.

#### Lessons learned from failures are equally instructive

Several AI-based tongue and pulse diagnosis models failed in cross-center validation due to sensor heterogeneity and subjective labeling noise. Similarly, some in silico predictions of synergistic herb pairs could not be reproduced experimentally because intestinal microbial metabolism of herbal glycosides was not modeled. These limitations underscore the necessity of embedding pharmacokinetic and metabolic constraints into AI pipelines.

## AI and TCM KGs: Structuring Complexity for Intelligent Discovery

The preceding sections have established NP as a powerful framework for mapping the molecular interactions of TCM and have detailed the transformative role of AI in enhancing this paradigm. While NP provides an invaluable network-based perspective on the “multicomponent, multitarget” nature of TCM, the inherent complexity, semantic richness, and multimodal nature of TCM knowledge necessitate a more powerful and expressive framework. The vast body of TCM knowledge, spanning millennia from ancient classics to modern clinical records, remains largely unstructured and fragmented, posing a substantial barrier to systematic analysis and integration. This section introduces the TCM KG as a paradigm-shifting technology that moves beyond associative networks to create a structured, machine-readable, and comprehensive representation of the entire TCM domain, thereby paving the way for a new era of intelligent discovery.

### From networks to knowledge graphs: A paradigm shift in structuring TCM knowledge

A TCM KG is formally defined as a large-scale semantic network that represents domain-specific knowledge as a structured collection of entities and their relationships [103]. Its basic unit is the triplet—consisting of a head entity, relation, and tail entity [104]. This triplet-based structure allows for the formal and unambiguous representation of complex facts, transforming unstructured information into a machine-readable format. A TCM KG integrates fragmented knowledge—from ancient texts to modern EHRs and biomedical databases—into a unified, queryable system that overcomes “knowledge islands” [103]. By doing so, it creates a comprehensive knowledge repository that supports advanced applications such as intelligent search, question-answering, and clinical decision support.

While both NP and KGs utilize graph structures, they differ fundamentally in scope, semantic richness, and purpose. The “herb–compound–target–disease” networks central to NP are best understood as specific, task-oriented graphs focused primarily on mapping putative biological interactions. They excel at generating hypotheses about the molecular mechanisms of herbal formulas. In contrast, a TCM KG is a far more generalized and semantically rich structure [105]. It represents a substantial expansion beyond the biological focus of NP. A full TCM KG incorporates not only herbs, compounds, targets, and diseases but also TCM-specific elements such as pattern, symptoms, properties, diagnostics, and theoretical principles.

This distinction reframes the relationship between the 2 paradigms. NP networks can be viewed as subgraphs or specific slices of a broader TCM KG. NLP-based extraction of relationships from literature and databases—central to NP—often yields structured KG-like data [7]. These KGs then provide the standardized and validated “data foundation” upon which robust NP analyses can be built [106]. This perspective positions the KG as the foundational knowledge layer, providing a stable, scalable, and semantically coherent backbone that enhances the reliability, scope, and reproducibility of NP studies.

The structured and integrated nature of KGs renders them uniquely capable of addressing several longstanding challenges that have impeded the modernization of TCM. First, in terms of overcoming data fragmentation and heterogeneity, TCM knowledge is scattered across diverse sources such as ancient texts, modern clinical guidelines, EHRs, and various biomedical databases [107]; KGs offer a robust framework to unify these heterogeneous sources, thereby establishing a single, coherent source of truth that connects classical wisdom with contemporary scientific data [1]. Second, regarding resolving terminological inconsistency and semantic ambiguity, the language of TCM is marked by nonstandardized expressions and semantic ambiguity—where the meaning of a term largely depends on context [108]; by defining a formal ontology, a KG standardizes terminology and clarifies semantic meaning, a process that makes the knowledge machine-readable and computationally tractable while reducing the risk of misinterpretation [1]. Third, in bridging ancient wisdom and modern science, a key advantage of the KG framework lies in its ability to formally link classical TCM concepts to modern biomedical entities; for instance, a KG can explicitly model the relationship between the TCM pattern “Blood Stasis” and the biological process of platelet aggregation, which creates a computational bridge enabling researchers to systematically investigate ancient theories using modern scientific methods and data.

### AI-powered knowledge extraction: The architectural blueprint of TCM KGs

Constructing a comprehensive and accurate TCM KG is a complex task that heavily depends on AI technologies. These technologies automate the extraction and structuring of knowledge from massive and diverse data sources. This section outlines the TCM KG construction pipeline, highlighting the role of AI in converting unstructured text into structured knowledge and the challenges involved [109]. The development of a TCM KG generally follows a systematic, multistage framework. It begins with data collection and preprocessing, drawing from TCM databases, biomedical databases, clinical guidelines, electronic medical records, and classical texts. Next is ontology design, in which experts define the KG schema—specifying entity types and the semantic relationships between them. The core of the pipeline is knowledge extraction, where AI models are applied to identify entities and their relationships from source texts. Finally, in the knowledge fusion and storage stage, extracted triplets are cleaned, deduplicated, and loaded into a graph database to enable efficient querying and analysis.

Recently, LLMs such as ChatGPT and ChatGLM have emerged as powerful tools for knowledge extraction [110]. By leveraging few-shot or even zero-shot learning, LLMs can perform named entity recognition and relation extraction with minimal task-specific training data. This substantially reduces reliance on large-scale, manually annotated datasets [109]. Advanced techniques include prompt engineering and the use of models such as Sentence-BERT to guide extraction. Sentence-BERT identifies semantically relevant prompts to enhance extraction accuracy [109].

**Fig. 6.**
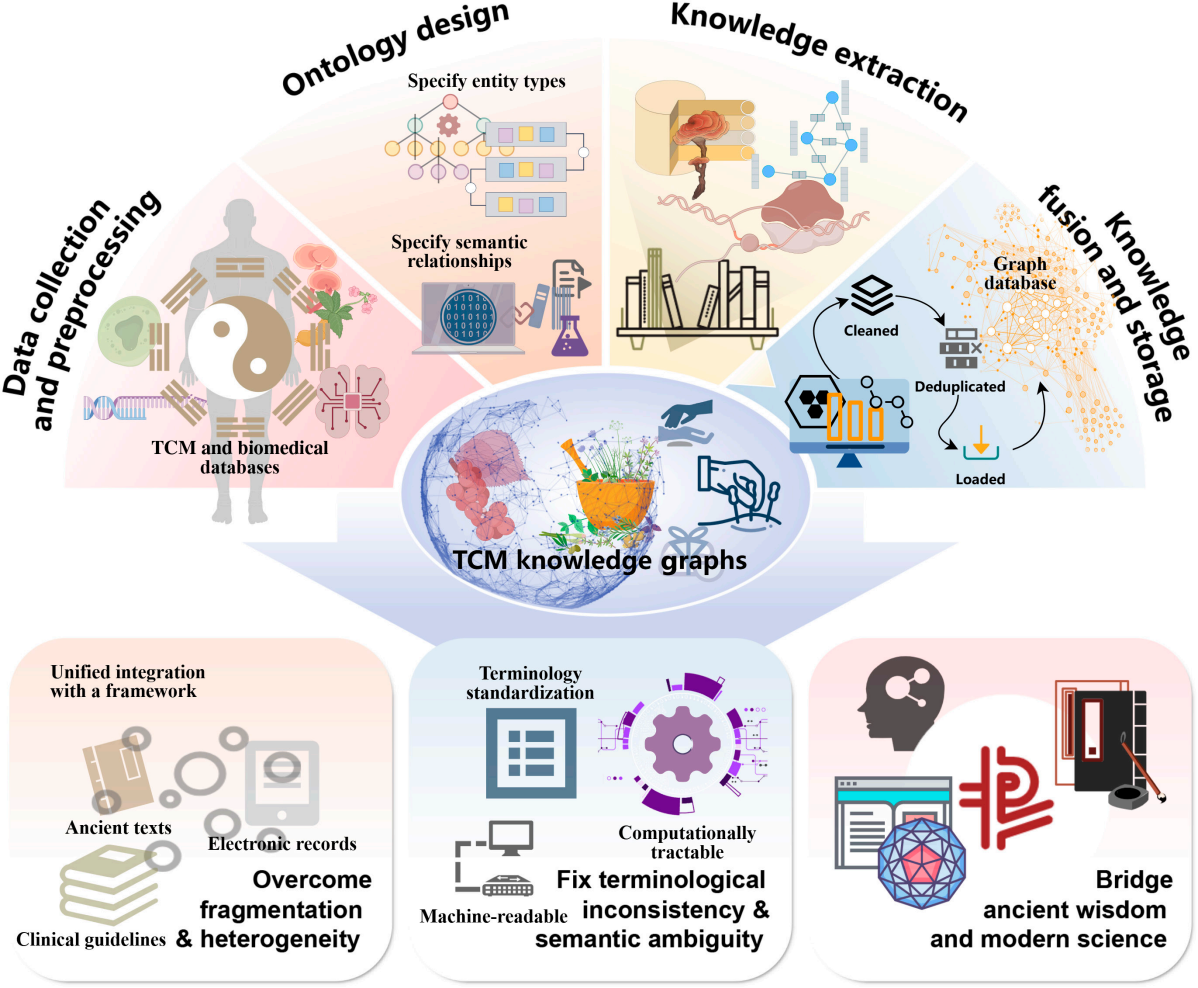
Schematic diagram of artificial intelligence (AI)-based construction method for traditional Chinese medicine (TCM) knowledge graph and its role in knowledge system optimization. The diagram systematically elucidates the AI-driven methodology for building TCM knowledge graphs and its transformative impact on TCM knowledge graph. The upper semicircular section of this schematic diagram outlines the technical workflow for constructing an AI-powered TCM knowledge graph. The process begins with the red module, representing data collection from TCM and biomedical databases and its subsequent preprocessing. This is followed by the orange module for ontology design, which defines the specific entities and their semantic relationships. The core of the workflow is the yellow module, where AI models perform knowledge extraction by identifying these entities and relationships. The final blue module completes the process through knowledge fusion and storage, which involves cleaning, deduplicating, and loading the data into a graph database. The lower section illustrates the impact of TCM knowledge graphs on TCM knowledge architecture. The left part tackles data fragmentation and heterogeneity by integrating diverse sources—such as ancient texts, clinical guidelines, and electronic records—into a unified framework. The middle part resolves terminology inconsistencies and semantic ambiguities by establishing standardized, machine-readable, and computable terms. Finally, the right part demonstrates the graph’s capacity to bridge ancient wisdom with modern science.

Despite their capabilities, LLMs present unique challenges. Their generative nature makes them prone to “hallucination”, where they may generate plausible but factually incorrect information or “overpredict” entities not present in the text [109]. This risk is particularly high in the TCM domain, as the complexity of TCM language, nonstandardized terminology, and subtle semantic differences can easily mislead general-purpose models [108]. A promising solution is a hybrid AI pipeline that leverages LLM strengths while mitigating their weaknesses. This pipeline incorporates a “self-validation” mechanism: After the initial extraction, the LLM is prompted a second time to verify its own output. For example, it might be asked, “Does the entity ‘*Ephedra sinica*’ (Mahuang) in the sentence belong to the entity type ‘Disease’? Answer Yes or No.” [109]. This self-correction loop functions as automated quality control, improving extraction reliability. This approach indicates that the future of high-quality medical KG construction does not lie in a fully automated, “hands-off” process, but in sophisticated human–AI collaboration. Humans define ontologies and validate outputs, while AI performs large-scale extraction and self-correction to build trustworthy, clinically relevant knowledge bases. Figure 6 clearly presents the complete construction process of the AI-driven TCM KG. Specifically, the upper section of Fig. 6 elucidates the technical pipeline from data collection to knowledge fusion, while the lower section demonstrates how this structured repository addresses data fragmentation and facilitates the bridge between ancient wisdom and modern clinical metrics. Within the proposed framework, Western clinical indicators serve both as node-level quantitative attributes and as hierarchical constraint signals. They are embedded into the KG to enrich phenotype representation while simultaneously guiding cross-layer alignment between molecular mechanisms and clinical manifestations through constraint-based regularization during model training (Fig. 6).

### Clinical implications: From structured knowledge to bedside decision support

The ultimate value of a TCM KG lies in its capacity to transform structured knowledge into actionable clinical intelligence [111]0. When coupled with AI inference, a KG evolves from a static repository into an active clinical decision support system that operates at the bedside [112]. In practical settings, this enables evidence-based personalized prescription [113], in which a clinician’s input of multimodal patient data—including symptoms, tongue and pulse signals, laboratory indices, and medication history—is mapped onto the KG to identify mechanistically plausible treatment strategies. Through AI-driven subgraph matching, the system retrieves herb combinations whose historical efficacy, molecular mechanisms, and pattern compatibility collectively best match the patient’s current state, thereby reducing empirical trial and error in prescription selection.

Beyond static decision support, the TCM KG further enables therapeutic trajectory prediction. By projecting a patient’s longitudinal clinical data onto historical treatment-response pathways embedded within the graph [114], AI models can anticipate disease progression, detect deviations from expected recovery patterns [115], and generate early warnings of adverse herb–drug interactions. This dynamic mapping transforms the KG into a learning clinical system that continuously refines its recommendations through real-world feedback. Collectively, these capabilities ensure that the structured complexity of TCM is not merely digitized, but computationally operationalized to improve diagnostic precision, treatment efficacy, and safety in modern clinical practice.

## Future Outlook: Deep Integration of AI and TCM

### AI and multiomics synergy

Rapid advances in genomics, proteomics, and metabolomics align with TCM’s multicomponent, multitarget therapeutic model. Integrating multiomics data offers new insights into TCM research, with AI playing a key role. AI, including ML and DL, can process complex biomedical data to uncover underlying patterns. In TCM, AI can help integrate genomics, proteomics, metabolomics, and other multilevel data to uncover the complex relationships between herbal components and human systems. For example, AI analysis can help identify the core active ingredients in TCM formulas, determine their targets, and construct “herb–compound–target–disease” networks [107]. This network-based approach reveals TCM mechanisms and supports new drug development. Furthermore, AI shows great potential in integrating multiomics data. Traditional biological research often focuses on analyzing data from a single layer, but the effects of herbal medicine typically involve multiple biological levels. Single-omics data cannot fully capture the mechanisms of action [18]. AI can integrate genomic, transcriptomic, proteomic, and metabolomic data to build system-level models and reveal TCM’s global regulatory effects [116]. For instance, AI can analyze multiomics data to predict the effects of herbal formulas on metabolic pathways and immune responses, providing new theoretical bases for comprehensive disease treatment.

### Data standardization and quality control enhancement

A substantial challenge in TCM research is data heterogeneity and standardization. Much of the data in traditional TCM literature and clinical practice lacks unified standards, making cross-study comparisons and data sharing difficult. Additionally, the individualized nature of herbal treatments presents another challenge—how to precisely control the quality of herbal medicines and ensure consistent therapeutic effects [117]. AI provides new solutions to these issues. Through ML and data mining techniques, AI can analyze data from diverse sources and standardize them. AI can help researchers extract useful information from large volumes of TCM literature, construct standardized databases, and apply natural language processing techniques to automate the analysis of TCM texts, extracting key information such as herb components, mechanisms of action, and clinical applications [118]. In terms of quality control, AI has demonstrated its power as well. Through multidimensional quality monitoring systems, AI can monitor the production process of TCM in real time, from raw material selection to production processes and final product quality checks, improving the consistency and efficacy of the products. For example, AI can use image recognition to assess the appearance quality of herbs and apply chemical analysis to quantitatively assess active ingredients, ensuring the consistency and therapeutic stability of TCM products [119].

### AI in personalized medicine

Personalized medicine is a key focus of modern medical research, and TCM’s principle of pattern differentiation provides a natural theoretical foundation for personalized treatment [120]. TCM emphasizes designing personalized treatment plans based on each patient’s specific condition, which aligns closely with the concept of precision medicine. However, traditional pattern differentiation relies on the physician’s experience and clinical judgment, often lacking systematic data support [121]. The introduction of AI can fill this gap, providing more precise guidance for personalized treatments through data analysis and intelligent models. With AI’s help, TCM can analyze a patient’s constitution, symptoms, tongue and pulse diagnosis, and other multidimensional data, combining the composition and mechanisms of herbal formulas to develop tailored treatment plans [105]. AI technology can also monitor the patient’s response to treatment in real time, adjusting the treatment strategy according to physiological changes and disease progression, maximizing the therapeutic effect [122]. In the future, AI will play an increasingly important role in precision treatment and personalized healthcare within TCM.

### Interdisciplinary collaboration and challenges in AI and TCM integration

While AI offers tremendous potential for the modernization of TCM, several challenges remain in achieving deep integration. First, AI applications in TCM require interdisciplinary collaboration, not only between computer science and TCM but also involving pharmacology, biology, clinical medicine, and other fields [123]. The application of AI in TCM requires close collaboration between TCM experts, AI technologists, and professionals from other relevant fields to push forward the modernization of TCM research [105]. Second, AI’s integration with TCM faces cultural and linguistic barriers. TCM’s theoretical system is deeply rooted in traditional Chinese culture, and many concepts and terms are difficult to accurately express in modern scientific language [124]. Therefore, AI research in TCM needs to carefully interpret traditional TCM theories, avoiding oversimplifying the complexity of TCM into a single biological model [125]. Moreover, the “black-box” issue of AI technology is also a concern. While AI models can provide efficient predictions and analysis, their internal mechanisms are often difficult to fully understand and explain. This may cause trust issues, particularly in a field like TCM, which is deeply rooted in tradition. Therefore, improving the transparency and interpretability of AI models to align with the theoretical framework of TCM will be a key issue during AI and TCM integration.

## Conclusion

TCM, characterized by its “multicomponent, multitarget, multipathway” therapeutic model rooted in holistic philosophy, has long been hindered by its complexity in aligning with modern scientific interpretation—especially given the mismatch between TCM’s systemic nature and the reductionist “one target, one drug” paradigm of Western pharmacology. However, the deep integration of AI with systems biology, particularly NP, has revolutionized TCM research: NP’s “herb–compound–target–disease” networks resonate with TCM’s holistic thinking, while AI addresses NP’s limitations—ML streamlines bioactive component screening and ADME/T prediction, DL decodes formula synergies, and the “computational prediction-experimental validation” workflow (validated in oncology, metabolic diseases, and infectious diseases) has become the gold standard for mechanism research. Advances in structural causal modeling and counterfactual reasoning provide methodological tools for small-sample causal inference, which is especially relevant for TCM clinical datasets characterized by high dimensionality but limited cohort size. Additionally, AI transforms TCM quality control by linking chemical signatures to efficacy, and integrates multiomics (genomics, proteomics, and metabolomics) to construct holistic regulatory networks, advancing TCM toward precision and comprehensiveness.

Despite these advancements, challenges persist: Data heterogeneity and lack of standardization risk “garbage in, garbage out” outcomes, DL’s “black box” limits interpretability, and neglect of TCM dosage compatibility disconnects models from clinical practice. Future progress will rely on unified data standards, interpretable AI algorithms, and integration of clinical dosage rules. Cutting-edge technologies like biological foundation models trained on large-scale protein sequences and molecular data, such as structure-aware language models, offer unprecedented opportunities to integrate herbal compound profiling with protein structural inference. Collectively, these developments signal a transition from static association mapping toward dynamic, causality-aware, and cross-scale biological logic reconstruction. Ultimately, AI is not just a tool for TCM modernization but a paradigm-shifting methodology—translating TCM’s holistic philosophy into modern scientific language, unraveling its efficacy mysteries, and enabling TCM to contribute more to global healthcare while informing modern multitarget drug development.

## Data Availability

The data have been made available in the manuscript.
